# Preparation and Evaluation of Novel Transfersomes Combined with the Natural Antioxidant Resveratrol

**DOI:** 10.3390/molecules24030600

**Published:** 2019-02-08

**Authors:** Pey-Shiuan Wu, Yu-Syuan Li, Yi-Ching Kuo, Suh-Jen Jane Tsai, Chih-Chien Lin

**Affiliations:** 1 Department of Cosmetic Science, Providence University, Taichung 43301, Taiwan; jwu2@pu.edu.tw (P.-S.W.); g1060037@gm.pu.edu.tw (Y.-S.L.); sin14990@gmail.com (Y.-C.K.); 2 Department of Applied Chemistry, Providence University, Taichung 43301, Taiwan; sjtsai@pu.edu.tw

**Keywords:** resveratrol, transfersome, natural antioxidant, edge activators, liposomal technique

## Abstract

Resveratrol (*tran*-3,5,4′-trihydroxystibene, RSV) is a kind of polyphenol which has anti-inflammatory, antioxidant, anti-allergy, and anti-cancer properties, as well as being a scavenger of free radicals and preventing cardiovascular diseases. However, it is quite unstable in light, heat, and other conditions, and decays easily due to environmental factors. For these reasons, this study used a new type of carrier, transfersome, to encapsulate RSV. Transfersome consists of phosphatidyl choline (PC) from a liposomal system and non-ionic edge activators (EA). EA are an important ingredient in the formulation of transfersome; they can enhance the flexibility of the lipid bimolecular membrane of transfersome. Due to its ultradeformability, it also allows drugs to penetrate the skin, even through the stratum corneum. We hope that this new encapsulation technique will improve the stability and enhance the permeability of RSV. Concluding all the tested parameters, the best production condition was 5% PC/EA (3:1) and 5% ethanol in distilled water, with an ultrasonic bath and stirring at 500 rpm, followed by high pressure homogenization. The optimal particle size was 40.13 ± 0.51 nm and the entrapment efficiency (EE) was 59.93 ± 0.99%. The results of antioxidant activity analysis showed that transfersomes were comparable to the RSV group (unencapsulated). During in vitro transdermal delivery analysis, after 6 h, D1-20(W) increased 27.59% by accumulation. Cell viability assay showed that the cytotoxicity of D3-80(W) was reduced by 34.45% compared with the same concentration of RSV. Therefore, we successfully prepared RSV transfersomes and also improved the stability, solubility, and safety of RSV.

## 1. Introduction

Resveratrol (*tran*-3,5,4′-trihydroxystibene, RSV), also known as polydatin, piceid, and grape polyphenol, is a kind of polyphenol that is present in plants. It is usually found in grape skin, mulberry, peanuts, and red wine. RSV has been shown to modulate the metabolism of lipids; it also has anti-inflammatory, antioxidant, anti-allergy, and anti-cancer properties, as well as being a scavenger of free radicals, anti-allergy, and preventing cardiovascular diseases, for example, and used in the prevention and treatment of atherosclerosis and hyperlipidemia [1,2]. Several studies have pointed out that RSV is an effective preventive medicine for cancers: It can inhibit the growth of tumors and thus help prevent cancers [3,4,5]. RSV belongs to the polyphenols. In terms of its antioxidant capacity, RSV has an aromatic ring structure as well as an OH group that provides electrons. The use of peroxides on unpaired electrons, including lipids, proteins, or free radical products generated in the electron transport chain that undergo oxygen-consuming reactions, can terminate resveratrol’s free radical chain reaction to reach its antioxidant capacity [6]. In addition to antioxidant activity, RSV has been found to have anti-inflammatory properties as well. According to previous animal- and cell-based studies, RSV exhibited the ability to inhibit the COX enzyme activity and further confirmed its anti-inflammatory mechanism [7,8]. 

Since RSV has poor water solubility [9], instability [10], and low bioavailability, its applications in cosmetics, foods, and drugs have been extremely limited. Therefore, the use of proper carriers may potentially improve the above problems. For example, liposome has been used in cosmetics for many years [11,12]. It has good compatibility with the skin, with no reported allergies, will degrade into phospholipids, and become part of the cell membrane. However, due to its tight structure, liposome lacks flexibility, which also affects the transdermal penetration of active compounds. 

In 1992, Cevc and Blume developed a novel transfersome consisting of a phosphatidyl choline (PC) and a non-ionic edge activator (EA), called “Transfersome” [13,14]. Phosphatidyl choline is the main component of the biomembrane, consisting of a hydrophilic polar head group of a phosphate group and two hydrophobic fatty acid chains. EA is a structure having both hydrophilicity and hydrophobicity, and a single chain surfactant with a large curvature is generally used, which destabilizes the lipid bilayer of the vesicles and increases the ultradeformability of the bilayer by lowering its interfacial tension. It also relatively affects the physical properties of the transfersome [15,16]. Therefore, it easily penetrates through skin pores much smaller than itself to achieve transdermal penetration and prolong the release and increase the activity of the drug [17,18,19]. However, until now, there have been no any studies focused on the development of RSV transfersome. 

Therefore, in this study, we used transfersome as a novel carrier to encapsulate resveratrol, through the testing of different ratios of PC/EA and ethanol content on the entrapment efficiency (EE), particle size distribution, and zeta potential of transfersome. It is expected that the encapsulation technique with the best preparation conditions will improve the stability of resveratrol, increase the penetration, and also reduce the cytotoxicity. 

## 2. Results

### 2.1. Characteristics of Transfersomes

In the study, we successfully prepared transfersomes using a high-pressure homogenization technique. Different edge activators, Tween 20, Plantacare^®^ 1200 UP, and Tween 80, were used to find out the proper formulation for RSV transfersome. In addition, several important parameters of the production process were also evaluated in the experiments. 

The results of the particle sizes, polydispersity index (PDI), and entrapment efficiency of transfersomes are shown in Table 1. The results of different proportions of PC/EA could show that the particle sizes of the A, B, and C groups were all less than 100 nm. Among them, the better entrapment efficiencies in the B group were 62.21% to 67%. At low EA concentrations (C group), EA was incorporated within the lipid bilayer according to the partition equilibrium between the aqueous and lipid phase, resulting in limited drug partition equilibrium caused by the production of fewer vesicles. Increasing EA-induced vesicle growth and imparting fluidity to the membrane bilayer resulted in improved EE (B group). Additionally, the pore generation due to a high concentration of EA led to leakage of the vesicles, resulting in high drug loss and, hence, low drug entrapment (C group). 

The zeta potential is a measurement of the surface potential of suspended particles. Particles with a zeta potential greater than ± 30 mV are considered to be stable, because the repulsive force of the same charge can avoid particle aggregation [20]. Table 2 shows that the zeta potential of the D group is smaller than that of the B group, probably because the ions in the buffer dissociate and affect the surface charge of the particles, and the D group is expected to have better stability. 

In addition, the particle sizes of D1-20(W) (64.28 nm) were the largest in the D group, due to the hydrophilic-lipophilic balance (HLB) value of the edge activator. In general, the lower HLB value makes the particle size smaller. Afterwards, the ethanol content was compared. Table 1 shows that the content of ethanol increased and particle size also enlarged, but the entrapment efficiency significantly dropped after two weeks. It might be caused by the fact that increasing the ethanol content reduces the stability of the membrane, and thus decreases the entrapment efficiency. Based on the above results, there is no significant difference between the entrapment efficiency of B1 (62.23%) and D1 (59.93%), but D1 had a smaller particle size (64.28 nm) and zeta potential (−19.53 mV) and, therefore, the following experiment was conducted in group D. 

### 2.2. Antioxidant Activity Assays

**Figure 1:**
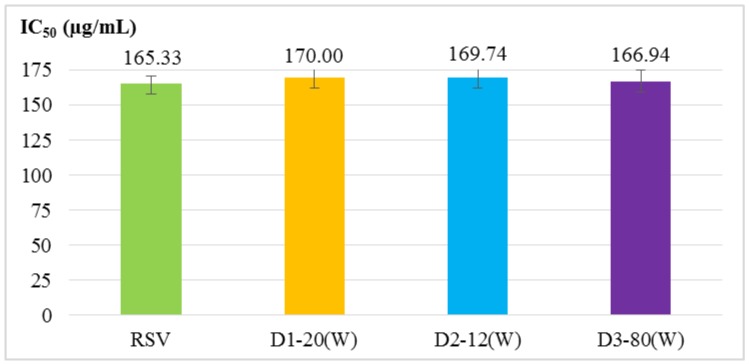
DPPH radical scavenging activity of RSV and RSV transfersomes (D1–20(W), D2–12(W), and D3–80(W) groups). The IC_50_ value is the sample to remove the 50% DPPH radicals (2.4 × 10^−4^ M) required concentration. The values are means ± SD.

**Figure 2:**
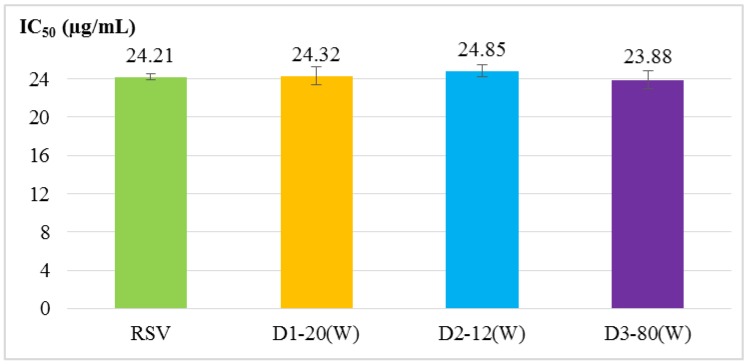
ABTS^+^ radical scavenging activity of RSV and RSV transfersomes (D1–20(W), D2–12(W), and D3–80(W) groups). The IC_50_ value is the sample to remove the 50% ABTS^+^ radical cations (7 × 10^−3^ M) required concentration. The values are means ± SD.

**Figure 3:**
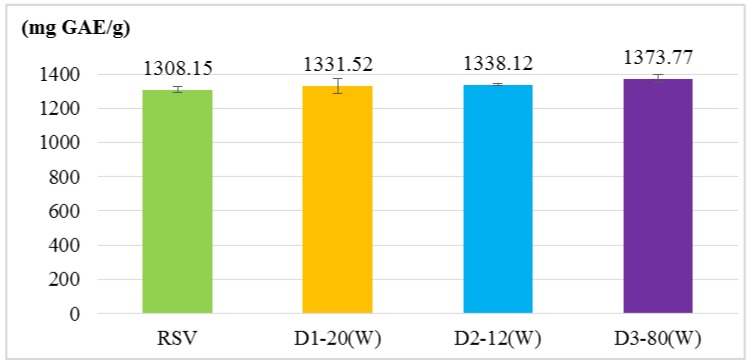
Total phenolic content of RSV and RSV transfersomes (D1–20(W), D2–12(W), and D3–80(W) groups). Total flavonoid content was measured by the Folin–Ciocalteu method and the results are presented as gallic acid equivalent (GAE). The 40 μL of sample was reacted with 40 μL of 2 N Folin-Ciocalteu reagent in a total volume of 600 μL. The values are means ± SD.

**Figure 4:**
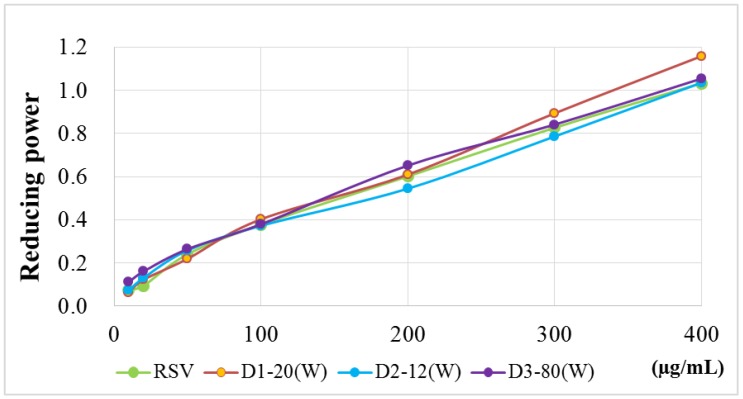
Reducing power of RSV and RSV transfersomes (D1–20(W), D2–12(W), and D3–80(W) groups). The ability of samples with various concentrations reduced 1% potassium ferricyanide (K_3_Fe(CN)_6_) is presented as the reducing power.

Four antioxidant activity experiments were used to evaluate RSV and RSV transfersomes, including α, α-diphenyl-β-picrylhydrazyl (DPPH) radical scavenging activity assay, 2,2′-azino-bis (3-ethylbenzothiazoline-6-sulphonic acid) (ABTS^+^) radical cation scavenging activity assay, total phenolic content and reducing power. The results (Figure 1, Figure 2, Figure 3 and Figure 4) show that the antioxidant activity of transfersomes was comparable to that of RSV, indicating that RSV antioxidant activity is not affected after coating. It was also proved that RSV did not lose its activity during the coating process, further confirming that the preparation method of this experiment was successful. 

### 2.3. In Vitro Transdermal Delivery Analysis

**Figure 5:**
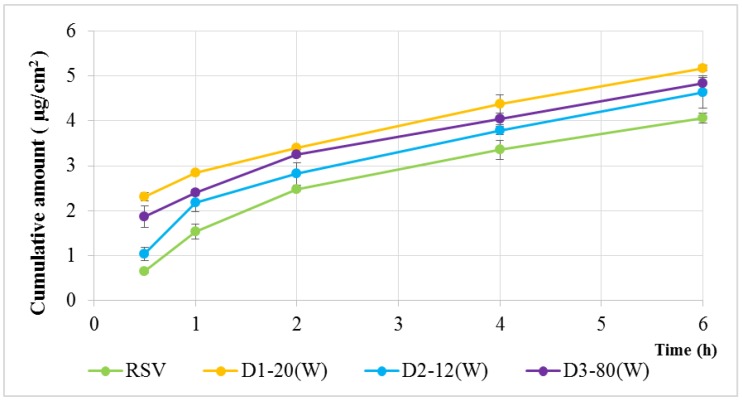
In vitro release profiles of RSV and RSV transfersomes (D1–20(W), D2–12(W), and D3–80(W) groups). The sample with 1.0 mL was placed in the donor site and 5.0 mL consisting of PBS and ethanol (70:30, *v*/*v)* was put into the receptor site. These sites were separated with a 0.785 cm^2^ Strat-M^®^ Membrane. The cumulative amount of RSV was analyzed in each time points.

In vitro drug release profiles are presented in Figure 5. It can be seen that the accumulative penetration of D1–20(W), D2–12(W), and D3–80(W) was higher than the unencapsulated RSV group (4.06 µg/cm^2^), and the highest accumulation of D1–20(W) was 5.18 µg/cm^2^ at 6 h. This shows that RSV can penetrate the skin more easily after encapsulation. This phenomenon was due to the addition of edge activators to the transfersomes, thereby increasing its ultradeformability and penetrability. Additionally, the penetration of D1–20(W) was higher than D3–80(W), the same type of edge activators, based on the carbon chain length. The carbon chain length of Tween 20 was shorter than Tween 80, so its higher ultradeformability made it easier for drugs to penetrate the skin. 

### 2.4. Cell Viability Assay

**Figure 6:**
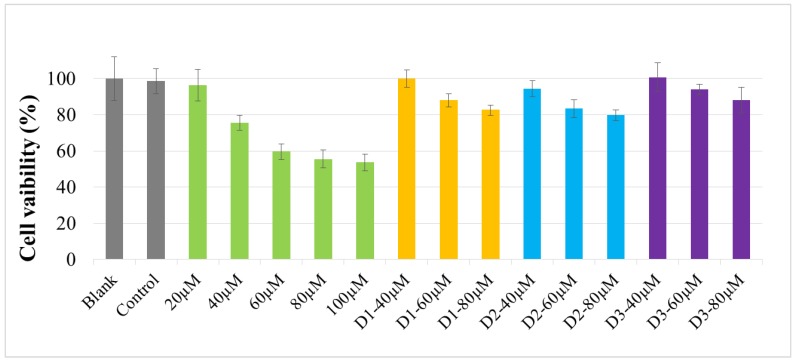
Cell viability of RSV and RSV transfersomes (D1–20(W), D2–12(W), and D3–80(W) groups). The cell viability of B16–F10 cells was analyzed by MTT assay after 24 h treatment. The values are means ± SD.

This study was conducted using different concentrations of RSV (20, 40, 60, 80, 100 μM) and transfersomes (40, 60, 80 μM). The cell viability (MTT assay) is shown in Figure 6. It was found that the cell viabilities of the transfersomes were all higher than 83%, among which D3-80(W) had the lowest cytotoxicity. Moreover, if the resveratrol concentrations of transfersomes are lower than 40 μM, almost no cytotoxicity is observed (data not shown). However, compared with the same concentration of RSV (40 to 80 μM), the cytotoxicity of transfersome was significantly reduced. The results demonstrate that RSV can diminish its cytotoxicity after the encapsulation; therefore, transfersomes are safer than RSV. 

## 3. Discussion

Resveratrol has poor water solubility, instability, and also low bioavailability; thus, its applications were restricted by these features. Additionally, there have not yet been any studies focused on the development of RSV transfersome. Therefore, it is important that we try to encapsulate the RSV into transfersome, which may have potential application for cosmetics, foods and drugs. 

In this study, we successfully prepared transfersomes using a high-pressure homogenization technique. Concluding the tested parameters, the best production condition was 5% PC/EA (3:1) and 5% ethanol in distilled water, with an ultrasonic bath and stirring at 500 rpm, followed by high-pressure homogenization (1500 bar). Therefore, these conditions were used to compare the three edge activators Tween 20, Plantacare^®^ 1200 UP. and Tween 80. 

According to the evaluation of particle size, entrapment efficiency and stability, the optimal particle size of D3–80(W) was 40.13 ± 0.51 nm. The entrapment efficiency of D1–20(W) was not significantly different from D3–80(W). The results of antioxidant activity analysis showed that there was no significant difference between the RSV transfersomes and RSV group (unencapsulated), indicating that RSV antioxidant activity is not affected after coating. Although the antioxidative activity of RSV transfersome is not enhanced by the encapsulation process, the solubility and stability might be improved by transfersome, and it is still supposed that the transfersome encapsulation is a good strategy for the application of RSV [21,22]. 

In the in vitro transdermal delivery analysis, after 6 h, D1–20(W) had the highest accumulation penetration, 5.18 μg/cm^2^, which was 27.5% more than that of the RSV group (Figure 5), confirming that transfersomes can significantly improve the transdermal delivery capacity of the active ingredient. The in vitro transdermal delivery analysis may simulate the situation of active compounds which be absorbed across the real skin. Therefore, this result also indicated that the application of RSV in the future might improve the bioavailability in many respects, including cosmetics, foods, and drugs. 

For the application of active ingredients in cosmetics and foods, and also in drugs, it is very important that safety should be the primary concern [23]. Our cytotoxicity assay showed that the cell viability of transfersome-treated cells improved, and the cytotoxicity of the D3–80(W) group decreased by about 34.45% when compared with the same concentration of RSV (Figure 6), demonstrating that transfersomes can reduce the cytotoxicity of RSV. Therefore, the above results prove that the transfersome encapsulation cannot only effectively increase the penetrating accumulation of resveratrol but also benefit its safety. 

## 4. Materials and Methods

### 4.1. Materials

Resveratrol was provided by Shaanxi Huifeng Pharmaceutical Co., Ltd. (Xi′an, China). Acetic acid and potassium hexacyanoferrate (K_3_Fe(CN)_6_) were purchased from Honeywell (Seelze, Germany). Dipotassium hydrogenphosphate (K_2_HPO_4_), potassium phosphate (KH_2_PO_4_) and sodium phosphate (Na_2_HPO_4_) were supplied by J.T. Baker^®^ (Phillipsburg, NJ, USA). Ethanol was obtained from Echo Chemical (Miaoli, Taiwan). Folin-Ciocalteu’s phenol reagent, trichloroethanoic acid (TCA), α, α-diphenyl-β-picrylhydrazyl (DPPH), and 3-(4,5-dimethyl-2-thiazolyl)-2,5-diphenyl-2H-tetrazolium bromide (MTT) were purchased from Sigma-Aldrich (St. Louis, MO, USA). Gallic acid was provided by MP BIOMEDICALS (USA). Iron trichloride (FeCl_3_) and sodium carbonate (Na_2_CO_3_) were obtained from Kanto Chemical Co., Inc. (Japan). Methanol was purchased from Aencore (Australia). Polysorbate 20 (Tween20) and polysorbate 80 (Tween80) were supplied by Croda (City, UK). 2,2’-azino-bis (3-ethylbenzothiazoline-6-sulphonic acid) (ABTS) was purchased from Tokyo Chemical Industry Co., Ltd. (Tokyo, Japan). Sodium phosphate monobasic (NaH_2_PO_4_) was provided by Choneye Pure Chemicals (Taipei, Taiwan). Kalium peroxodisulfate (K_2_S_2_O_8_) was obtained from Acros Organics (Morris Plains, NJ, USA). Lauryl glucoside (Plantacare^®^ 1200 UP) was provided by BASF (Ludwigshafen, Germany). Lecithin (Emulmetik^TM^ 900) was purchased from Lucas Meyer Cosmetics (Champlan, France). 

### 4.2. Preparation of Transfersome

First, different proportions of lecithin and edge activators (Table 3) were uniformly dispersed in PBS buffer (pH 6.8) [24] or distilled water containing various contents of alcohol for 3 h with ultrasonic shaking by an ultrasonic bath (DC 300H, DELTA, New Taipei City, Taiwan) and stirring at 500 rpm. Second, the mixture underwent intermittent ultrasonic shaking (2 s on, 2 s off). The mixture was homogenized in five cycles with a high-pressure homogenizer (APV-2000, SPXFLOW, UK) at 1500 bar. Finally, samples were stored in a dark room at 25 °C [25]. 

### 4.3. Particle Sizes and Zeta Potential of Transfersome

Dynamic light scattering (DLS) is one of the standard methods for measuring particle sizes in fluids. This method is based on the examination of random particle movement due to constant Brownian motion [26]. Particle size and zeta potential were assessed by DLS and electrophoretic light scattering (ELS) using a dynamic light scattering nanoparticle size analysis (Malvern Zetasizer Nano ZS, Malvern Instruments Ltd., Malvern, UK). 

### 4.4. Entrapment Efficiency of Resveratrol

The sample was centrifuged at 10,000 rpm for 15 min, and the supernatant was diluted with methanol. The sample was filtered by Phree^TM^ Phospholipid Removal (Phenomenex, Torrance, CA, USA) to remove impurities and phospholipids and determine the concentrations of RSV by HPLC (Pump: V6815, UV–VIS: V6830, KNAUER, Berlin, Germany). RSV separation was carried out on a C18 column (Kinetex 5u EVO C18 100A 250 × 4.6 mm, Phenomenex, Torrance, CA, USA) using a mobile phase consisting of methanol containing 0.5% acetic acid in water (80:20, *v*/*v*) at a flow rate of 0.8 mL/min. The detection wavelength was set at 310 nm. 

The RSV EE was calculated by Equation (1) [27]:

(1) $$\mathrm{EE}\;\left(\%\right)=\left(\frac{\mathrm{total}\;\mathrm{RSV}\;\mathrm{amount}-\mathrm{free}\;\mathrm{RSV}\;\mathrm{amount}}{\mathrm{total}\;\mathrm{RSV}\;\mathrm{amount}}\right)\times 100$$

### 4.5. Stability of Transfersome

The transfersome samples were stored at 25 °C and dark for 14 days. Then the stability was evaluated by its entrapment efficiency, particle size, and PDI. 

### 4.6. Antioxidant Activity Evaluation

#### 4.6.1. DPPH Radical Scavenging Activity Assay

When the sample reacted with DPPH, hydrogen was added to inhibit the oxidation chain reaction. At this time, the DPPH solution changed its color from purple to yellow. This experiment used the IC_50_ comparison sample to remove the 50% DPPH required concentration. Twenty microliters of varying concentrations of sample solution were added to 180 μL of 2.4 × 10^−4^ M DPPH solution. The mixing solutions were incubated in dark at room temperature for 30 min, and the absorbance at 517 nm was recorded by an ELISA-reader (MQX-200, BioTek, Winooski, VT, USA). All determinations were carried out in triplicate.

The radical scavenging rate was calculated by Equation (2) [28]:

(2) $$\mathrm{DPPH}\;\mathrm{scavenging}\;\mathrm{rate}\left(\%\right)=\left(\frac{A0-\mathrm{Ai}}{A0}\right)\times 100$$

where A0 is the absorbance of blank, and Ai is the absorbance of the sample.

#### 4.6.2. ABTS^+^ Radical Cation Scavenging Activity Assay

The peroxidase catalyzes the oxidation reaction between ABTS^+^ and H_2_O_2_, forming a stable blue-green water-soluble ABTS^+^ cation radical. When the ABTS^+^ cation radical is reduced by an antioxidant or combined with another radical, the absorbance value decreases or disappears. Twenty microliters of varying concentrations of sample solution were added to 180 μL of 7 × 10^−3^ M ABTS^+^ solution. The mixing solutions were incubated in the dark at room temperature for 10 min, and the absorbance at 734 nm was recorded by an ELISA-reader (MQX-200, BioTek, Winooski, VT, USA). All determinations were carried out in triplicate. 

The radical scavenging rate was calculated by Equation (3):

(3) $${\mathrm{ABTS}}^{+}\mathrm{radical}\;\mathrm{cation}\;\mathrm{scavenging}\;\mathrm{rate}\left(\%\right)=\left(\frac{A0-\mathrm{Ai}}{A0}\right)\times 100\%$$

where A0 is the absorbance of blank; Ai is the absorbance of the sample.

#### 4.6.3. Total Phenolic Content

Total flavonoid content was measured by the Folin–Ciocalteu method. The phenolic acid compounds have easily oxidizable hydroxyl groups, and phosphomolybdic acid in the Folin-Ciocalteu reagent will oxidize it and reduce itself to form a blue compound. In our study, the calibration curve was established using gallic acid as a standard. To the 40 μL of sample, we added 520 μL of distilled water and 40 μL of 2 N Folin-Ciocalteu reagent, and this was shaken for 6 min. Afterwards, we added 400 μL of 7% Na_2_CO_3_ and incubated it in the dark at room temperature for 90 min; the absorbance at 750 nm was recorded by an ELISA-reader (MQX-200, BioTek, Winooski, VT, USA). The total phenol content of the sample could be determined by comparing the calibration curve of gallic acid (GAE). All determinations were carried out in triplicate.

#### 4.6.4. Reducing Power

Antioxidants reduced potassium ferricyanide (K_3_Fe(CN)_6_) to potassium ferrocyanide (K_4_Fe(CN)_6_), and potassium ferrocyanide reacted with Fe3^+^ to form Prussian blue. Three hundred microliters of varying concentrations of sample solution were added to 300 μL of 0.2 M potassium phosphate buffer (pH 6.6) and 300 μL of 1% K_3_Fe(CN)_6_ for 20 min at 50 °C. After cooling to room temperature, 300 μL of 10% TCA was added and centrifuged at 3000 rpm for 20 min. The supernatant was added to 500 μL of distilled water and 100 μL of 0.1% FeCl_3_. The mixing solutions were incubated in the dark for 10 min and the absorbance at 700 nm was recorded by an ELISA-reader (MQX-200, BioTek, Winooski, VT, USA). All determinations were carried out in triplicate. 

The reducing power was calculated by Equation (4):

(4) Reducing power=Ai−A0

where A0 is the absorbance of blank; Ai is the absorbance of the sample.

### 4.7. In Vitro Transdermal Delivery Analysis

The transdermal drug delivery system has been widely used in the human body to absorb in vitro release. The release of transfersomes was tested by a Franz type diffusion cell (Logan FDC-6, Somerset, NJ, USA). The cell consisted of two compartments, the donor and the receptor sections. These sections were separated with a 0.785 cm^2^ Strat-M^®^ Membrane (Merck, Darmstadt, Germany) and hydrated with PBS (pH 7.4) and ethanol (70:30, *v*/*v*) [17] for 30 min. A 1.0 mL transfersome was placed in the donor site and 5.0 mL consisting of PBS and ethanol (70:30, *v*/*v*) was poured into the receptor site. The Franz type diffusion cells were stirred at 36.7 ± 0.3 °C with magnetic stirrers. Aliquots of 300 µL were withdrawn at various time intervals (0.5, 1, 2, 4, 6 h) and replaced by the same volume of fresh medium. The amount of released RSV was passed through a 0.45 μm polytetrafluoroethylene (PTFE) membrane filter, and the amount of released RSV was measured by high-performance liquid chromatography. 

### 4.8. Cell Culture and Cell Viability Assay

The cell line used in this experiment was B16-F10 (BCRC 60031) mouse melanoma cells purchased from the Food Industry Research and Development Institute (Hsinchu, Taiwan). B16-F10 cells were seeded at a density of 6 × 10^3^ cell/well in a 96-well plate and maintained in 100 µL Dulbecco’s modification of Eagle medium (DMEM) supplemented with 10% PBS for 24 h in a humidified incubator (37 ^O^C, 5% CO_2_). Each well was then treated with RSV and RSV transfersomes at serial concentrations. After being cultured for 24 h, 100 µL (1.0 mg/mL) of MTT solution was added into each well for 30 min to allow the formation of formazan crystal. Subsequently, supernatant was removed carefully and 100 µL DMSO was added to each well. Cell viabilities were evaluated using an ELISA-reader (MQX-200, BioTek, Winooski, VT, USA) at 540 nm. 

### 4.9. Statistical Analysis

All tests were performed in triplicates. The values are presented as means ± SD of three replicates. The statistically significant differences were evaluated by the Student’s test.

## 5. Conclusions

Our results demonstrate that transfersome improves the instability, solubility, bioavailability, and safety of RSV. For the in vitro transdermal delivery, D1-20(W) had the highest cumulative amounts. For cell viability, the D3–80(W) group had the lowest cytotoxicity. The results indicated that the RSV transfersomes D1–20(W) and D3–80(W) are the best groups. Therefore, applications of RSV transfersomes in the fields of cosmetics, foods and drugs will be considered as a potential formulation in the future. 

## Figures and Tables

**Table 1:** Entrapment efficiency (EE) and particle sizes of transfersomes.

-	Particle Size (nm)		PDI		EE (%)	
-	0 Days	14 Days	0 Days	14 Days	0 Days	14 Days
A1–20(B)	48.34 ± 0.19	49.27 ± 0.47	0.145 ± 0.007	0.166 ± 0.005	39.76 ± 2.70	25.19 ± 1.26
A2–12(B)	56.35 ± 0.31	58.53 ± 0.66	0.128 ± 0.016	0.119 ± 0.018	33.31 ± 0.87	24.85 ± 2.27
A3–80(B)	64.53 ± 0.41	66.32 ± 0.30	0.042 ± 0.011	0.041 ± 0.003	48.27 ± 2.29	30.30 ± 0.43
B1–20(B)	72.03 ± 0.27	73.37 ± 0.16	0.072 ± 0.017	0.074 ± 0.012	62.23 ± 3.65	52.01 ± 0.45
B2–12(B)	55.46 ± 0.55	62.43 ± 1.04	0.222 ± 0.013	0.197 ± 0.038	62.21 ± 1.56	56.60 ± 0.51
B3–80(B)	58.48 ± 0.19	57.90 ± 0.11	0.304 ± 0.010	0.270 ± 0.016	67.96 ± 1.85	60.17 ± 0.81
C1–20(B)	49.85 ± 0.14	50.02 ± 0.35	0.127 ± 0.013	0.111 ± 0.014	43.50 ± 0.96	26.72 ± 1.99
C2–12(B)	73.72 ± 15.54	60.56 ± 2.66	0.233 ± 0.085	0.268 ± 0.006	42.06 ± 2.00	24.70 ± 1.21
C3–80(B)	68.85 ± 1.77	67.10 ± 0.27	0.424 ± 0.044	0.373 ± 0.003	44.00 ± 0.56	31.70 ± 1.88
D1–20(W)	64.28 ± 0.60	66.43 ± 0.21	0.206 ± 0.008	0.177 ± 0.004	59.93 ± 0.99	50.60 ± 1.33
D2–12(W)	43.07 ± 0.21	61.67 ± 1.93	0.312 ± 0.039	0.557 ± 0.024	56.13 ± 1.52	47.82 ± 0.54
D3–80(W)	40.13 ± 0.63	45.67 ± 0.45	0.266 ± 0.009	0.201 ± 0.009	59.01 ± 1.02	51.17 ± 1.72
E1–20(W)	81.39 ± 0.41	67.56 ± 0.21	0.146 ± 0.009	0.097 ± 0.008	61.69 ± 0.29	31.76 ± 0.74
E2–20(W)	75.88 ± 0.59	73.03 ± 0.74	0.119 ± 0.008	0.095 ± 0.022	56.71 ± 0.86	24.66 ± 2.29

**Table 2:** Zeta potential of transfersomes.

-	Zeta (mV)		Zeta (mV)
B1-20(B)	−1.93 ± 0.24	D1-20(W)	−19.53 ± 0.91
B2-12(B)	−6.29 ± 0.47	D2-12(W)	−59.90 ± 0.20
B3-80(B)	−0.54 ± 0.08	D3-80(W)	−23.93 ± 0.31

**Table 3:** Changeable experimental parameters to affect transfersome.

	Composition(%*W*/*V*)							
No.	RSV	Emulmetik 900	Tween-20	1200UP	Tween-80	Ethanol	PBS Buffer	Distilled Water
A1-20(B)	0.2	3.33	1.67			5.00	q.s. to 100	
A2-12(B)	0.2	3.33		1.67		5.00	q.s. to 100	
A3-80(B)	0.2	3.33	-	-	1.67	5.00	q.s. to 100	
B1-20(B)	0.2	3.75	1.25			5.00	q.s. to 100	-
B2-12(B)	0.2	3.75		1.25		5.00	q.s. to 100	
B3-80(B)	0.2	3.75	-	-	1.25	5.00	q.s. to 100	
C1-20(B)	0.2	4.00	1.00			5.00	q.s. to 100	-
C2-12(B)	0.2	4.00		1.00		5.00	q.s. to 100	
C3-80(B)	0.2	4.00	-	-	1.00	5.00	q.s. to 100	-
D1-20(W)	0.2	3.75	1.25			5.00		q.s. to 100
D2-12(W)	0.2	3.75		1.25		5.00		q.s. to 100
D3-80(W)	0.2	3.75	-	-	1.25	5.00	-	q.s. to 100
E1-20(W)	0.2	3.75	1.25			10.00	-	q.s. to 100
E2-20(W)	0.2	3.75	1.25	-	-	20.00	-	q.s. to 100
